# Integrated Analysis of miRNA and mRNA Expression in Childhood Medulloblastoma Compared with Neural Stem Cells

**DOI:** 10.1371/journal.pone.0023935

**Published:** 2011-09-09

**Authors:** Laura A. Genovesi, Kim W. Carter, Nicholas G. Gottardo, Keith M. Giles, Peter B. Dallas

**Affiliations:** 1 Brain Tumour Research Program, Telethon Institute for Child Health Research, Centre for Child Health Research, University of Western Australia, Perth, Australia; 2 Division of Bioinformatics, Telethon Institute for Child Health Research, Centre for Child Health Research, University of Western Australia, Perth, Australia; 3 Department of Haematology and Oncology, Princess Margaret Hospital for Children, Perth, Australia; 4 Laboratory for Cancer Medicine, University of Western Australia Centre for Medical Research, Western Australian Institute for Medical Research, Perth, Australia; Roswell Park Cancer Institute, United States of America

## Abstract

Medulloblastoma (MB) is the most common malignant brain tumor in children and a leading cause of cancer-related mortality and morbidity. Several molecular sub-types of MB have been identified, suggesting they may arise from distinct cells of origin. Data from animal models indicate that some MB sub-types arise from multipotent cerebellar neural stem cells (NSCs). Hence, microRNA (miRNA) expression profiles of primary MB samples were compared to CD133+ NSCs, aiming to identify deregulated miRNAs involved in MB pathogenesis. Expression profiling of 662 miRNAs in primary MB specimens, MB cell lines, and human CD133+ NSCs and CD133− neural progenitor cells was performed by qRT-PCR. Clustering analysis identified two distinct sub-types of MB primary specimens, reminiscent of sub-types obtained from their mRNA profiles. 21 significantly up-regulated and 12 significantly down-regulated miRNAs were identified in MB primary specimens relative to CD133+ NSCs (p<0.01). The majority of up-regulated miRNAs mapped to chromosomal regions 14q32 and 17q. Integration of the predicted targets of deregulated miRNAs with mRNA expression data from the same specimens revealed enrichment of pathways regulating neuronal migration, nervous system development and cell proliferation. Transient over-expression of a down-regulated miRNA, miR-935, resulted in significant down-regulation of three of the seven predicted miR-935 target genes at the mRNA level in a MB cell line, confirming the validity of this approach. This study represents the first integrated analysis of MB miRNA and mRNA expression profiles and is the first to compare MB miRNA expression profiles to those of CD133+ NSCs. We identified several differentially expressed miRNAs that potentially target networks of genes and signaling pathways that may be involved in the transformation of normal NSCs to brain tumor stem cells. Based on this integrative approach, our data provide an important platform for future investigations aimed at characterizing the role of specific miRNAs in MB pathogenesis.

## Introduction

Medulloblastoma (MB) is the most common malignant pediatric brain tumor, with an incidence of approximately 0.5 per 100 000 children less than 15 years of age [1]. Current multi-modal treatment consists of surgery, radiation and adjuvant chemotherapy [2], [3]. Although recent advances in therapy have lifted five-year survival rates for average-risk MB to 80%, the outcome for high-risk patients remains poor [4], [5]. Additionally, the long term consequences of existing treatment protocols can be profound and include both intellectual and developmental impairment [6]. Clearly, less invasive and more effective treatment strategies for MB are urgently required.

MB is a heterogeneous disease and current risk stratification strategies often fail to accurately predict disease outcome. Recent microarray gene expression studies and genomic analyses have contributed to the improved sub-classification of MB, incorporating clinical and demographic characteristics to identify at least four distinct sub-types according to their specific gene expression signatures [7]–[10]. Sub-types A and B are characterized by over-active Wingless-type MMTV integration site family (Wnt) and Hedgehog (Hh) signaling respectively, while sub-types C and E exhibit enriched expression of genes associated with neuronal differentiation and photoreceptor differentiation, respectively [8]. A fifth sub-type (D) was also identified, characterized by a mixed signature of both neuronal and photoreceptor differentiation. Sub-type specific gene expression signatures may ultimately prove critical for the development of new targeted therapeutic agents as well as the identification of subsets of patients responsive to specific targeted therapies.

The identification of several molecular sub-types of MB suggests that different sub-groups may arise from distinct cells of origin. Indeed, accumulating evidence from animal models suggests that some MB sub-types arise from cerebellar granule cell precursors (GCPs), multipotent cerebellar neural stem cells (NSCs) [11]–[13], or Zic1+ precursor cells of the dorsal brainstem [14]. In human MB, a subpopulation of CD133-expressing cells was identified that displayed similar properties to normal NSCs, including self-renewal and multipotency [15], [16]. Additional studies demonstrated that these putative brain tumour stem cells (BTSCs) were capable of initiating tumor formation in immunodeficient mice [17]. Engraftment of as few as 100 CD133+ cells was sufficient for tumor formation, while as many as 100 000 CD133− cells failed to produce tumors. Taken together, these findings in both humans and mice strongly implicate normal CD133+ NSCs as candidate cells of origin for MB. While mRNA expression profiling studies suggest that specific MB sub-types may be derived from normal NSCs (Bertram *et al*, manuscript in preparation); the analysis of non-coding RNA expression represents another approach to test this hypothesis. Integrated expression profiling of both coding and non-coding RNAs will further characterize the molecular sub-types of MB, and may give further insight into MB pathogenesis and putative cells of origin.

microRNAs (miRNAs) are a class of short, non-coding RNAs that post-transcriptionally down-regulate gene expression by binding to the 3′-untranslated region (UTR) of protein coding transcripts, resulting in either mRNA cleavage or translational repression [18], [19]. At the time of writing, more than 1,000 human miRNA genes have been identified (miRBase Release 16) [20] and recent studies investigating miRNA target site conservation within human 3′UTRs suggest that expression of >60% of human protein coding genes may be regulated by miRNAs [21]. An individual miRNA may potentially bind and regulate many different mRNA targets with related function, such as those belonging to a single cell signaling pathway [22], whilst a given mRNA target may be regulated by several different miRNAs, creating a highly complex combinatorial network of gene regulation [23]. miRNAs have important roles in the regulation of diverse cellular processes including development, cell proliferation, apoptosis, differentiation and metabolism [24]–[28]. Not surprisingly, abnormalities in miRNA expression and function have been shown to affect these normal physiological processes, with increasing evidence supporting a critical role for deregulated miRNA expression in cancer initiation and progression [29], [30].

We hypothesized that the identification of miRNA-regulated networks of gene expression in human primary MB specimens, relative to CD133+ NSCs, will improve our understanding of MB pathogenesis. To date, several research groups have investigated deregulated miRNA expression in MB [31]–[36], including comparisons to normal human adult and fetal cerebellum [33], [34], [36] which represent heterogeneous tissues at different developmental stages. Our approach is distinct but complementary to these reports, and is the first to assess miRNA expression levels in primary human MB specimens and MB cell lines in comparison to human CD133+ NSCs and CD133− neural progenitor cells (NPCs). To identify candidate miRNA-regulated networks of gene expression that may be involved in MB pathogenesis, we then integrated these miRNA expression profiles with mRNA gene expression data we obtained from the same samples. Furthermore, we validated the regulation of a set of predicted target genes by specific miRNAs *in vitro*. In summary, our findings suggest that these miRNAs may have potential as both therapeutic targets and clinical biomarkers of MB.

## Methods

### Ethics statement

Written approval to undertake this study was obtained from the Princess Margaret Hospital (PMH) human ethics committee. Written consent to use tumor material for research purposes was obtained from the parents of patients according to PMH ethics committee guidelines. All tumor material was de-identified to ensure patient anonymity.

### Patient samples

Primary brain tumors were collected at PMH, embedded in optimal cutting temperature compound (OCT) and snap-frozen. Ten MB specimens were analyzed. The age of patients ranged from seven months to 13 years, with a mean age of 4.5 years. Gender distribution was seven males to three females (2.3∶1) (Table 1).

**Table 1:** Clinical data and methods used to profile miRNA and mRNA expression.

Sample	Gender	Age	M Status	miRNA array	mRNA gene array
M1	Female	9	M0	Yes	No
M2	Female	3	Unknown	Yes	Yes
M6	Male	7	M+	Yes	Yes
M7	Male	1.5	M0	Yes	Yes
M9	Male	2	Unknown	Yes	No
M11	Male	5	M+	Yes	Yes
M14	Female	3	M0	Yes	Yes
M15	Male	3	M+	Yes	Yes
M17	Male	4	M+	Yes	Yes
M22	Male	1	Unknown	Yes	No

Metastatic status was defined as M0 (no metastasis) and M+ (distant metastases).

### Cell lines and neurosphere maintenance

The desmoplastic MB cell line PER547 and classic MB cell lines PER 568 and PER 621 were established from primary specimens [37] and cultured in RPMI supplemented with 20% fetal calf serum at 37°C with 5% CO_2_. Primary MB specimens from which these cell lines were derived were not included in the primary MB cohort profiled in this study. Human NSCs propagated as neurospheres were derived from human ESC lines hES3 (WiCell Research Institute, Madison, WI, USA) and MEL1 (Australian Stem Cell Centre, Melbourne, Australia) using protocols described previously [38], [39]. Neurospheres were maintained in neural basal medium, supplemented with N2 (Gibco, Mt Waverley, Australia), Penicillin/Streptomycin (Gibco, Mt Waverly, Australia), L-Glutamine (Sigma, Castle Hill, Australia), Interferrin-Transferrin-Selenium (Gibco, Mt Waverley, Australia), EGF (20 ng/ml, Sigma, Castle Hill, Australia) and bFGF (20 ng/ml, Chemicon, Melbourne, Australia). Neurosphere cultures were propagated by mechanical splitting 1∶2–1∶4 [40] and cultured in ultra-low adherent 96 well plates (Corning, Melbourne, Australia) with growth factor supplementation every 2–3 days.

### Neurosphere dissociation and CD133+ NSC isolation

Neurospheres were dissociated using the trypsin-neural dissociation kit (Miltenyi Biotec, North Ryde, Australia) as described previously [41]. Dissociated NSC suspensions were washed and re-suspended in FACS buffer (1xPBS supplemented with 0.5% BSA (Sigma, Castle Hill, Australia) and 2 mM EDTA (Sigma, Castle Hill, Australia). Cells were blocked in Fc block (Miltenyi Biotec, North Ryde, Australia) for three min at room temperature and CD133+ NSCs were isolated as described [41]. FACs enrichment of CD133− NPCs was 100% for both hES3 and MEL1 ESC lines, with the enrichment of CD133+ NSCs 81.1% and 97% for the hES3 and MEL1 ESC lines, respectively.

### Small RNA isolation and enrichment

RNA enriched for small RNAs was isolated from primary specimens, cell lines and NSC/NPCs using the miRNeasy mini kit (Qiagen, Melbourne Australia), according to the manufacturer's protocol. Briefly, OCT compound was mechanically removed from embedded frozen primary tumor specimens and tumor tissue was placed in pre-weighed 1.5 ml eppendorf tubes containing QIAzol reagent. For MB cell lines, up to 1×10^7^ cells were harvested and pelleted by centrifugation for 5 min at 8000 rpm, prior to RNA extraction. On-column DNase digestion was utilized for all samples with RNase-free DNase (Qiagen, Melbourne, Australia), according to manufacturer's recommendations. RNA quality was assessed by 1.5% agarose gel electrophoresis and quantified on a Nanodrop 1000 (Biolab, Scoresby, Australia). RNA quantity and purity was estimated by the ratio of absorbance at 260 nm and 280 nm (OD_260_∶OD_280_), with ratios between 1.8 and 2.0 being considered optimal.

### miRNA expression profiling

miRNA profiling was performed using quantitative real-time RT-PCR utilizing pre-printed Taqman low density assay (TLDA) microfluidic cards (Human miR v2.0, Applied Biosystems, Melbourne, Australia). Each TLDA card set contained MGB-labeled probes specific to 762 mature miRNAs plus six endogenous small nucleolar RNAs (MammU6, RNU44, RNU48, RNU24, RNU43, RNU6B) for data normalization and relative quantification. Briefly, reverse transcription was performed with 30 ng small RNA enriched RNA using Megaplex RT stem loop primer pools (Life Technologies, Melbourne), Multiscribe Reverse Transcriptase (Life Technologies, Melbourne), RNase inhibitor and100 nM deoxynucleotide triphosphates (dNTP). Two multiplex pools were used per sample, each pool containing reagents and primers for 384 mature miRNAs and small nucleolar RNAs. The multiplexed RT and Megaplex pre-amplification reactions were performed according to manufacturer's instructions. Pre-processing of raw TLDA data files consisted of threshold and baseline corrections for each sample, with each amplification plot assessed to confirm that the threshold cycle (Ct) value corresponded with the midpoint of logarithmic amplification (SDS 2.3, Life Technologies, Melbourne). Ct values greater than 32 were imputed to 32 according to the technical recommendation. Raw miRNA expression data is available from the Telethon Institute for Child Health Research (TICHR) webserver: http://bioinformatics.childhealthresearch.org.au/datasets/.

### Endogenous control (EC) gene normalization for miRNA gene expression analysis

To determine the most appropriate combinations of genes for data normalization, the expression stability and abundance of candidate endogenous control genes were assessed using the BestKeeper software program [42]. To assess the range of expression levels of candidate EC genes, threshold cycle (C_t_) values for each were plotted across all samples (Figure S1). Subsequently, Bestkeeper was employed to evaluate the expression stability of several candidate EC genes, whilst simultaneously assessing the best possible combination of EC gene pairs for relative quantification of miRNA expression (Table S1). Ranking of the candidates was performed based upon deviation of gene expression, displayed as correlation of coefficient (r) to the Bestkeeper Index (BI), defined as the geometric mean of all candidate EC genes. Based on the abundance and stability of candidate EC genes, *MammU6* and *RNU48* were selected for the normalization of miRNA expression data, using the equation 2^−ΔCt^, where ΔCt = (Ct_miR_−Ct_mean endogenous control_).

### Statistics and Bioinformatics

All analyses were performed utilizing log_2_(2^−ΔCt^) transformed normalized miRNA expression data. Unsupervised agglomerative hierarchical clustering of miRNA expression profiles was performed using Euclidean distance and pairwise Pearson correlations with complete linkage. Results were visualized using dendrograms and heatmaps. All statistical analyses were performed in R statistical environment version 2.9.2 [43]. Visualization of clustering analyses was carried out using gplots R package (http://cran.r-project.org/web/packages/gplots/index.html). Principle component analysis (PCA) was performed using companion to applied regression (CAR) package in R (http://cran.r-project.org/web/packages/car/index.html). To identify specific miRNAs that were differentially expressed between MB primary specimens, MB cell lines and normal NSC/NPC samples, a two sample *t*-test was performed utilizing the normalized means (log_2_(2^−ΔCt^) of expression levels for each miRNA in MB primary specimens, cell lines, CD133+ NSCs and CD133− NPCs. For CD133+ NSCs and CD133− NPCs, normalized means (log_2_(2^−ΔCt^) of expression levels were obtained by averaging the expression of individual miRNAs from both hES3 and MEL1, therefore representing a pool of two ESC lines. Significantly deregulated miRNAs were defined as those having a p<0.01 and a differential expression value >log_2_ fold change (FC) of three (eight C_t_s). False discovery rate (FDR) corrected p-values were also calculated. For comparison analyses, venn diagrams were generated in R using limma package.

### Integration of miRNA and mRNA expression data

The integration of miRNA and mRNA expression data was performed using the online tool microRNA and mRNA integrated analysis (MMIA) [44]. Multiple integration analyses were performed, utilizing input lists including both significantly up- and down-regulated miRNAs identified in primary MB specimens from miRNA profiling analysis (Table S2). The miRNA target prediction algorithm TargetScan 5.1 was employed for all analyses [21], [45], [46], with miRNA and mRNA combined analysis performed as previously described [47]. To refine miRNA-mRNA target interactions, mRNA expression data were log_2_ transformed and a mean two-fold cut-off (fch <−2, >2) for each target gene in primary MB specimens was employed for mRNA data analysis. Due to a limited sample size, we were unable to perform FDR testing on the integration itself (as provided by MMIA). To limit potential false positive findings, we utilized a strict *t*-test p-value cutoff of <0.01 in combination with a log_2_ differential expression FC of greater than three for the deregulated miRNAs (when compared to CD133+ NSCs). Only a subset of MB primary specimens for which both miRNA and mRNA profiles were available was included in these analyses (Table 1). Ingenuity pathway analysis (IPA; Ingenuity Systems) was employed to assign biological function to putative target genes of miRNAs with significantly altered expression, utilizing candidate miRNA-mRNA target gene pairs for which upon integration an inverse correlation of expression was observed. IPA output was focused upon canonical pathway genesets and ranked based on statistical significance.

### Validation of miR-935 and miR-10a target gene regulation in MB

Candidate miR-935 target genes obtained from MMIA analyses, where miRNA and mRNA expression was inversely-correlated, were ranked according to TargetScan context score, with a context score greater than or equal to −0.4 subsequently selected as the cut-off for validation in the laboratory. miR-935 expression levels were obtained using Taqman qRT-PCR miRNA assay (Life Technologies) and correlated to selected putative target gene levels using Pearson's correlation. To validate selected mRNAs as targets of a given miRNA, miRNAs were over-expressed by transient transfection of synthetic precursor miRNAs (pre-miRs) at 5 nmol/L (Life Technologies) in PER-547 cells and 30 nmol/L (Life Technologies) in PER-568 cells using Ribofect transfection reagent (Bioline). Following small RNA-enriched RNA extraction, significant up-regulation of miR-935 and miR-10a was confirmed by qRT-PCR using two way analysis of variance (ANOVA) and an unpaired t-test, respectively. Following total RNA extraction (Qiazol), target mRNA gene expression levels were measured by reverse transcriptase quantitative PCR (qRT-PCR) at 24 hours post-transfection using Quantifast SYBR Green Master Mix (Qiagen) with the primers: *ACTB* (5′-CAT GTA CGT TGC TAT CCA GGC -3′ (forward) and 5′- CTC CTT AAT GTC ACG CAC GAT -3′ (reverse); *APC* (5′- CCT CAT CCA GCT TTT ACA TGG C-3′ (forward) and 5′- CGC CTG CCT CTC TTG TCA T-3′ (reverse); *KIAA0232* (5′- CTG CTG TCC AGT GTC TTC GAT-3′ (forward) and 5′- GGG AGA CCC CTC TAA CTT TTT GT-3′ (reverse); *MYT1* (5′- GGA CGC CTC TGT TTC GGA TG-3′ (forward) and 5′- ATC CAA AAT GGG ACT TGA CGG-3′ (reverse); *RELN* (5′- TCC GGG ACA AGA ATA CCA TGT-3′ (forward) and 5′- CCA AAT CCG AAA GCA CTG GAA-3′ (reverse); *SLC5A3* (5′- CCC AAT TTA CAT CCG GTC AGG-3′ (forward) and 5′- ATA CAG ATC CAC CGA GAG CTT-3′ (reverse); *TBC1D9* (5′- CCG TCA GGG TTG GAT GTA CC-3′ (forward) and 5′- GAA GAA ATG CTC ACT GGA CCG-3′ (reverse) and *ZFAND6* (5′- ACA GCC AAG TGC CTA TGC TTT-3′ (forward) and 5′- CAG ACA GAC TAC TGA CAG AGG T-3′ (reverse). *PIK3CA* mRNA levels were normalized to β-actin mRNA using Taqman assays on demand (Life Technologies), according to manufacturer's recommendations.

PIK3CA protein levels were analyzed by Western blotting at 48, 72 and 96 h post-transfection. The anti-pan actin and PI3 Kinase p110α monoclonal antibodies were purchased from NeoMarkers and Cell Signaling, respectively.

### mRNA gene expression profiling

Total RNA was extracted from primary MB specimens, MB cell lines, CD133+NSCs and CD133− NPCs and subsequently labelled and hybridized to Affymetrix HG-U133A arrays as described previously [48]. Unsupervised hierarchical clustering analysis was performed on the primary MB specimens, MB cell lines, CD133+ NSCs, and CD133− NPCs using the expression levels of 20 of the 24 genes (LEF1, RUNX2, DCX, MAB21L1, PTCH1, PDLIM3, NEUROG1, DLL3, PDGFA, FOXG1B, GRM1, VAMP4, CDKN1C, SERPINF1, NRL, CRX, NMNAT2, SMARCD3, GABRA5 and DCC) that classify MB molecular subgroups A–E as defined by Kool et al [8]. The genes OTX2, LEMD1, PTPN5 and ZNF179 were omitted from the analysis because they are not represented on the Affymetrix HG-U133A array.

## Results

### Global miRNA expression analyses of primary MB specimens, MB cell lines and normal NSCs revealed four distinct clusters

**Figure 1:**
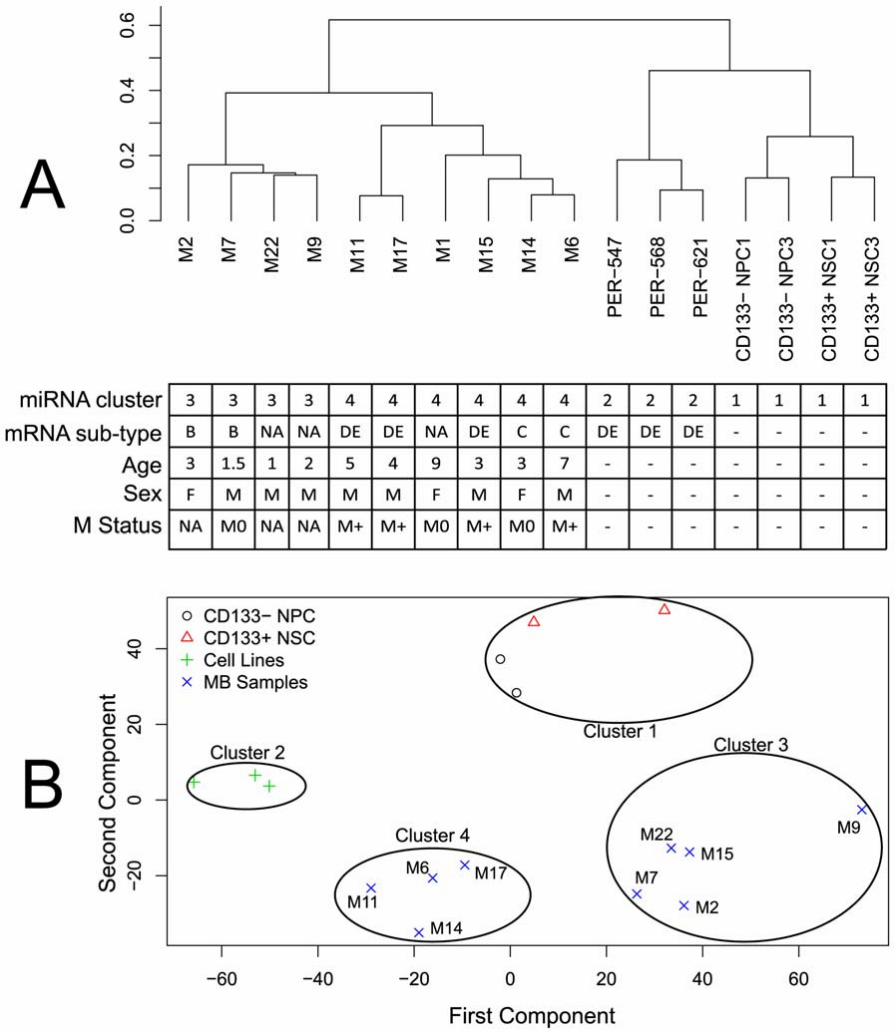
Clustering analysis of primary MB, MB cell lines and CD133+ NSCs and CD133− NPCs. (**A**) **Unsupervised hierarchical clustering** and (**B**) **PCA**. The analyses were based on normalized expression data for 662 miRNAs in ten primary MB specimens, three MB cell lines (PER-547, PER-568 and PER-621), two CD133+ NSC and two CD133− NPC samples. Sample numbers refer to those in Table 1. The similarity metric utilized for unsupervised hierarchical clustering was Pearson's correlation (r). MB sub-typing determined from mRNA gene expression analysis is indicated. Sub-type B is characterized by over-active SHH signaling, whilst sub-type C is characterized by the enriched expression of genes associated with neuronal differentiation. Sub-type E is characterized by enriched expression of photoreceptor genes, whilst sub-type D is characterized by mixed neuronal and photoreceptor genes. Primary MB specimens defined as “NA” were not available for sub-typing analysis. The M1 primary sample failed on the Pool B TLDA card and was therefore excluded from PCA, as this type of analysis cannot be performed on incomplete datasets. Primary MB sample, M15, grouped with cluster three, as compared to cluster four in Figure 1A.

**Figure 2:**
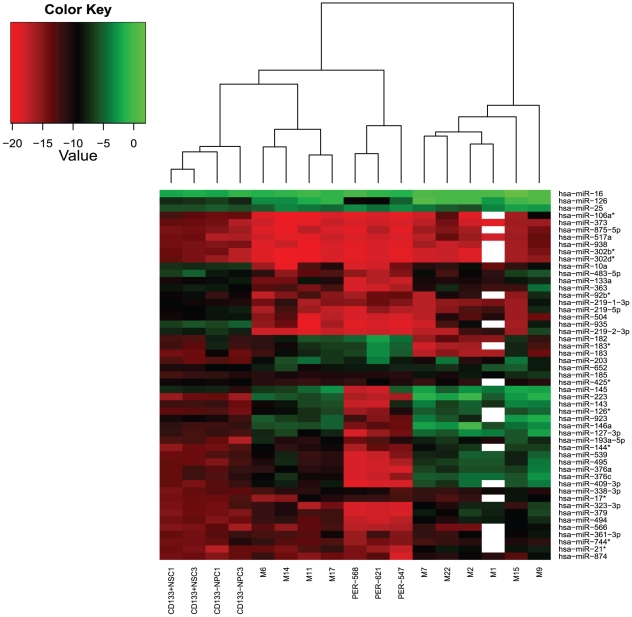
Heatmap analysis based on deregulated miRNAs in MB primary specimens and cell lines compared to CD133+ NSCs. Sample numbers refer to those in Table 1. The similarity metric utilized for this analysis was log_2_(2^−ΔCt^) transformed miRNA values obtained from qRT-PCR profiling analysis. miRNA expression in normal CD133+ NSCs was determined from averaging log_2_(2^−ΔCt^) transformed miRNA values of CD133+ NSCs from both hES3 and MEL1 ESC lines. A red to green color scale (−20 to +2) depicts normalized miRNA expression on a log scale, with median expression across all samples represented as black. White spaces in the heatmap are due to M1 primary sample failing on Pool B TLDA card.

miRNA profiles were generated for primary MB specimens, MB cell lines, CD133+ NSCs and CD133− NPCs using qRT-PCR. Unsupervised hierarchical clustering analysis of the 663 miRNAs revealed distinctive expression patterns for MB primary specimens, NSCs, NPCs and MB cell lines. The dendrogram included two main branches representing four clusters of samples (Figure 1A). Cluster one consisted of CD133+ NSC and CD133− NPC samples, which were distinct from both primary specimens and MB cell lines. MB cell lines comprised cluster two, with PER-547 separate to PER-568 and PER-621. Interestingly, primary MB samples segregated into two distinct clusters (cluster three and cluster four), suggesting two sub-types of primary MB specimens based upon their miRNA profiles. Principal component analysis (PCA) was applied to provide an additional method of unsupervised visualization of miRNA expression profiles. This revealed the same four clusters identified in the initial hierarchical clustering (Figure 1B). Spatial separation of CD133+ NSCs and CD133− NPCs from MB primary specimens and cell lines was evident. MB cell lines appeared as a separate cluster, with MB primary samples segregated into two distinct groups. Additional clustering analysis based on the 50 most significantly differentially expressed miRNAs in both MB primary specimens and cell lines compared to CD133+NSCs was consistent with these findings (Figure 2) (For miRNA list see Table S2).

### Up-regulation of miRNAs mapping to chromosomal region 14q32 in MB

Paired *t*-tests were employed to identify differences in individual miRNA expression levels between primary MB specimens and normal CD133+ NSCs and CD133− NPCs. Of the 663 miRNAs included in the analysis, we identified 33 miRNAs (p<0.01) that were significantly differentially expressed in primary MB samples in comparison to CD133+ NSCs, including 21 of 33 (63.6%) that were up-regulated and 12 of 33 (36.3%) that were down-regulated. Lists of significantly up- and down- expressed miRNAs in MB were generated on the basis of p-values for each miRNA (Table 2). Interestingly, many of the up-regulated miRNAs mapped to identical chromosomal regions. In particular, 10 of the 21 (47.6%) up-regulated miRNAs mapped to chromosomal region 14q32 (Table 2). miRBase (Release 16) reported 58 miRNAs located at 14q32, thus 33.3% of miRNAs we have identified as deregulated in MB map to this region (Fisher's exact test, p<0.001). In addition, four of the 21 (19%) up-regulated miRNAs were located at chromosomal region 17q. A similar analysis was performed to identify 53 differentially expressed miRNAs in MB versus CD133− NPCs, the majority of which were up-regulated in MB (Table S3). Of these, 16/53 (30%) were also found to be differentially expressed when compared to CD133+ NSCs (Figure S2).

**Table 2:** Significantly up- and down-regulated miRNAs in primary MB specimens relative to CD133+ NSCs.

miRNA	Chromosomal location	p value	FDR adjusted p value	Fold Change (log2)
**UP-REGULATED**				
hsa-miR-127-3p	14q32.2	0.0001	0.0071	6.33
hsa-miR-539	14q32.31	<0.0001	0.0031	5.91
hsa-miR-495	14q32.31	<0.0001	0.0031	6.00
hsa-miR-409-3p	14q32.31	0.0001	0.0058	7.10
hsa-miR-494	14q32.31	0.0012	0.0316	3.80
hsa-miR-376c	14q32.31	0.0014	0.0341	6.39
hsa-miR-379	14q32.31	0.0019	0.0400	4.90
hsa-miR-376a	14q32.31	0.0073	0.1209	5.04
hsa-miR-323-3p	14q32.31	0.0077	0.1248	3.45
hsa-miR-203	14q32.33	0.0004	0.0132	5.96
hsa-miR-193a-5p	17q11.2	0.0001	0.0048	3.93
hsa-miR-144*	17q11.2	0.0069	0.1167	6.12
hsa-miR-21*	17q23.1	0.0025	0.0509	3.67
hsa-miR-338-3p	17q25.3	0.0043	0.0807	3.36
hsa-miR-143	5q32	<0.0001	0.0010	6.06
hsa-miR-145	5q32	0.0016	0.0359	3.67
hsa-miR-146a	5q34	<0.0001	0.0010	7.57
hsa-miR-126	9q34.3	<0.0001	0.0005	6.28
hsa-miR-126*	9q34.3	<0.0001	0.0005	6.61
hsa-miR-223	Xq12	0.0052	0.0906	11.16
hsa-miR-361-3p	Xq21.2	0.0009	0.0236	3.06
**DOWN-REGULATED**				
hsa-miR-483-5p	11p15.5	0.0091	0.1408	−4.91
hsa-miR-10a	17q21.32	0.0007	0.0219	−5.19
hsa-miR-373	19q13.42	<0.0001	0.0031	−3.74
hsa-miR-935	19q13.42	0.0002	0.0082	−8.37
hsa-miR-92b*	1q22	0.0050	0.0888	−4.32
hsa-miR-302b*	4q25	0.0005	0.0153	−3.84
hsa-miR-302d*	4q25	0.0049	0.0888	−4.11
hsa-miR-219-1-3p	6p21.32	0.0002	0.0082	−4.86
hsa-miR-219-5p	6p21.32/9q34.11	0.0008	0.0236	−4.93
hsa-miR-219-2-3p	9q34.11	0.0003	0.0127	−7.12
hsa-miR-106a*	Xq26.2	0.0100	0.1507	−3.98
hsa-miR-504	Xq26.3	0.0003	0.0106	−5.02

miRNAs are sorted according to their chromosomal location. miRNA expression in normal CD133+ NSCs was determined from averaging log_2_(2^−ΔCt^) transformed miRNA values of CD133+ NSCs from both hES3 and MEL1 ESC lines.

### Integrative analysis of miRNA/mRNA expression identifies several putative regulatory networks in MB

We performed MMIA to identify putative miRNA-regulated networks in MB. Interestingly, these integrative analyses revealed several miRNA mapping to 14q32 that were predicted to have identical target genes. Of particular interest, up-regulated miRNAs miR-494 and miR-495 were predicted to target *CTNND2*, while up-regulated miR-376c and miR-409-3p were predicted to target *NR2F2*, which were both down-regulated in primary MB specimens relative to CD133+ NSCs (Table S4).

To address the functional significance of the putative targets of differentially expressed miRNAs, IPA enrichment analysis was performed focusing on dysregulated mRNA targets of both the up- and down-regulated miRNAs identified as differentially expressed in MB (Table S4 and Table S5, respectively). The top 30 enriched pathways in primary MB specimens relative to CD133+ NSCs and CD133− NPCs are listed in Table S6 and Table S7, respectively. Functional enrichment analyses of negatively-correlated targets of both up- and down-regulated miRNAs revealed 106 significant pathways (Fisher's exact test, p<0.05). This included over-representation of pathways associated with neuronal migration and nervous system development, such as Axonal Guidance (p<0.0001) and Reelin Signaling in Neurons (p<0.0001) (Table 3), and pathways related to cell proliferation and programmed cell death including the Insulin growth factor 1 (IGF1) (p = 0.0002) and Phosphatase and tensin homolog (PTEN) pathways (p = 0.0005) (Table 3). Common miRNA target genes of these over-represented pathways included both Phosphoinositide-3-kinase, catalytic alpha polypeptide (*PIK3CA*) and the related Phosphoinositide-3-kinase, regulatory subunit 1 (*PIK3R1*) featuring in 77 of the 106 (72.6%) significant pathways. Similarly, Adenomatosis polyposis coli (*APC*) featured in 12 of the 106 (11.3%) signaling pathways. Thus, pathway analysis suggested deregulated miRNA-regulated networks of gene expression in MB affect both developmental signaling and the regulation of cell proliferation.

**Table 3:** Pathway enrichment analysis of putative mRNA targets of deregulated miRNAs in primary MB specimens versus CD133+ NSCs using IPA pathway curated gene sets.

Ingenuity Canonical Pathways	p value	Genes
Molecular Mechanisms of Cancer	<0.0001	NF1, PIK3R1, APC, ARHGEF3, WNT5A, PRKAR2A, PRKD1, NOTCH1, SMAD3, PIK3CA, TCF3, CCND2, SMAD2, RALGDS, FZD7, ARHGEF12, TGFBR2, RRAS2, RAP2A, PRKACB, NRAS, CDC25A, MAPK10
Colorectal Cancer Metastasis Signaling	<0.0001	PIK3R1, APC, WNT5A, PRKAR2A, SMAD3, PIK3CA, TCF3, SMAD2, RALGDS, MSH6, FZD7, TGFBR2, RRAS2, PRKACB, NRAS, GNB5, BIRC5, MAPK10
Ovarian Cancer Signaling	<0.0001	PIK3R1, APC, FZD7, RRAS2, WNT5A, PRKAR2A, TCF3, PIK3CA, NRAS, PRKACB, CD44, MSH6
Reelin Signaling in Neurons	<0.0001	PIK3R1, ARHGEF12, ARHGEF3, ITGA6, PIK3CA, RELN, DCX, ARHGEF9, MAPK10
Axonal Guidance Signaling	<0.0001	PIK3R1, GLI3, WNT5A, PRKAR2A, PRKD1, PIK3CA, WASL (includes EG:8976), EPHA3, SEMA5A, NFATC1, FZD7, EFNB3, ARHGEF12, RRAS2, NFAT5, EPHB2, PRKACB, NRAS, GNB5, NTRK2, SHANK2
IGF-1 Signaling	0.0002	PIK3R1, RRAS2, PRKAR2A, PIK3CA, GRB10, NRAS, PRKACB, FOXO3, CYR61
Human Embryonic Stem Cell Pluripotency	0.0002	PIK3R1, APC, FZD7, FGFR2, TGFBR2, WNT5A, SMAD3, TCF3, PIK3CA, SMAD2, NTRK2
PPARα/RXRα Activation	0.0003	NCOR2, RRAS2, TGFBR2, PRKAR2A, MED1, SMAD3, NRAS, PRKACB, MAP4K4, SMAD2, ACOX1, GHR
Regulation of IL-2 Expression in Activated and Anergic T Lymphocytes	0.0003	NFATC1, RRAS2, TGFBR2, NFAT5, SMAD3, NRAS, SMAD2, MAPK10
PTEN Signaling	0.0005	PIK3R1, FGFR2, RRAS2, TGFBR2, PIK3CA, NRAS, FOXO3, NTRK2, GHR

The top ten enrichment terms are listed and were sorted based on statistical significance.

### Validation of putative miR-935 and miR-10a targets in MB

To evaluate the robustness of miRNA/mRNA predictions identified by the integration of miRNA and mRNA expression data and to gain insight into the potential role of differentially expressed miRNAs in MB, we first focused on the most significantly down-regulated miRNA, miR-935. Several putative miR-935 targets predicted by TargetScan were up-regulated in MB compared to CD133+ NSCs (Table S5). Among these were genes with established roles in neuronal migration, cell adhesion and the development of the CNS including Reelin (*RELN*) and Myelin transcription factor 1 *(MYT1)*. We ranked up-regulated predicted target genes on the basis of Targetscan context score and selected the top six candidates for functional testing, in addition to *APC* due to its presence in multiple enriched pathways. To assess whether putative miRNA target genes had inverse expression to miR-935 levels in MB, we correlated expression levels of miR-935 with selected target genes in MB primary specimens. A moderate to strong negative correlation was observed for the majority of miR-935 target genes, supporting prediction and integration analyses (Table 4).

**Table 4:** Pearson's correlation analysis between miR-935 relative expression levels and expression levels of selected target genes.

Target Gene	Pearson's value (r)	p-value
*APC*	0.04	0.4597
*KIAA0232*	−0.23	0.3196
*MYT1*	0.84	0.0096
*RELN*	−0.39	0.1886
*SLC5A3*	−0.66	0.0527
*TBC1D9*	−0.35	0.2219
*ZFAND6*	−0.55	0.0985

Pearson's correlation (r) indicates a moderate to strong negative correlation value for majority of target genes.

**Figure 3:**
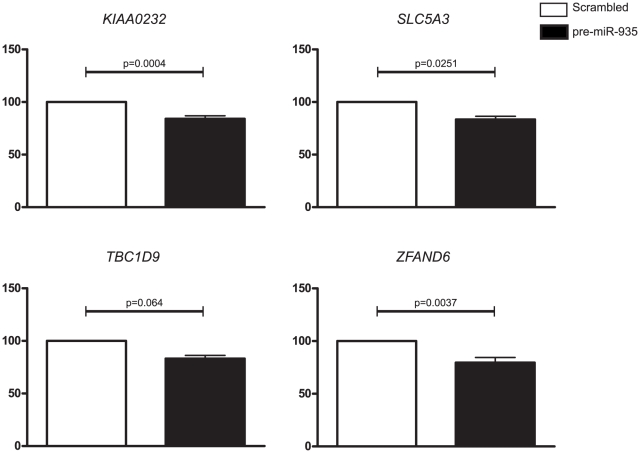
Validation of putative miR-935 targets in PER-547 cells. Quantitative RT-PCR analysis of (**A**) *KIAA0232* (**B**) *SLC5A3* (**C**) *TBC1D9* and (**D**) *ZFAND6* expression levels in PER-547 cells 24 h after transfection with scrambled negative control or pre-miR-935. Target gene expression levels in pre-miR-935-transfected samples were normalized to scrambled control levels. p values were obtained from a two way ANOVA on log transformed expression data. Error bars represent mean +/− SEM from six independent experiments.

To address whether miR-935 regulates predicted target genes in MB, transient over-expression of miR-935 in the MB cell line, PER-547, which has 7000 fold decreased levels of miR-935 relative to normal CD133+ NSCs, was performed. Significant up-regulation of miR-935 was confirmed by qRT-PCR, with ∼11 000 fold up-regulation of miR-935 in pre-miR-935 transfected cells at 24 h compared to scrambled negative control cells (p<0.01). Significant down-regulation of three of the seven predicted miR-935 target genes was observed at the mRNA level (Figure 3A–D): Solute carrier family 5 member 3 (*SLC5A3*) (p = 0.0251), Hypothetical protein LOC9778 (*KIAA0232*) (p = 0.0004) and Zinc Finger AN1-type domain 6 (*ZFAND6*) (p = 0.0037). Furthermore, down-regulation of TBC domain family member 9 (*TBC1D9*) approached statistical significance (p = 0.064). Expression levels of *APC*, *RELN* and *MYT1* remained unchanged at the mRNA level following transfection (data not shown), although it is possible that miR-935 could regulate expression of *APC*, *RELN* and *MYT1* at the translational level. Following this, we investigated the potential regulation of *PIK3CA* by miR-10a, given the over-representation of this gene in the majority of enriched signaling pathways. Transient over-expression of miR-10a in the MB cell lines, PER-547 and PER-568 was performed, which has 125 fold and 2200 fold decreased levels of miR-10a relative to normal CD133+ NSCs, respectively. Significant up-regulation of miR-10a was confirmed by qRT-PCR, with ∼40 000 fold (p = 0.005) up-regulation of miR-10a in pre-miR-10a transfected PER-547 and ∼200 000 fold up-regulation in PER-568 cells at 24 h, compared to scrambled negative control cells. However, transient over-expression of miR-10a in both MB cell lines, PER-547 and PER-568, did not affect PIK3CA mRNA or protein levels (data not shown). Nevertheless, our results with mir-935 target genes indicated that the integration of miRNA and mRNA expression data was a useful approach for the identification of putative miRNA-regulated genes in MB.

## Discussion

The precise cell of origin for MB remains to be determined, however data from both human and mice studies suggest that some MB sub-types arise from NSCs [11]–[13], [15]–[17]. Several research groups have investigated deregulated miRNA expression in MB pathogenesis and have evaluated miRNA expression levels relative to normal human cerebellum. However, normal cerebellum consists of a heterogenous population of cell types and may not represent the optimal normal control tissue [33], [34], [36]. This is the first study to perform global miRNA profiling of human MB primary specimens relative to a putative cell of origin, CD133+ NSCs.

miRNA profiling has been used previously to classify tumors according to their molecular and histological sub-type [49]–[51]. In this study, clustering analysis based on miRNA expression profiling identified two distinct sub-groups of primary MB specimens, a finding reminiscent of sub-types identified on the basis of mRNA expression profiles. Primary MB specimens in cluster three represented Shh-driven sub-type B, whilst specimens in cluster four represented sub-types C, D and E (Bertram et al., manuscript in preparation and [8]). Additionally, our clustering analysis based on miRNA expression profiling revealed MB cell lines clustering as a distinct subgroup from primary MB specimens. This is consistent with previous reports in bladder cancer [52], cholangiocarcinoma [53] and germ cell tumors [54] highlighting underlying differences in miRNA expression between immortalized cell lines grown in culture and primary tumor specimens, in addition to sample heterogeneity due to the absence of stroma in cell lines. Although based on small sample sizes, our findings suggest that miRNA expression profiling is capable of classifying primary MB specimens into different tumor sub-groups. Larger sample numbers are required to further characterize these sub-groups at the miRNA level.

The majority of differentially expressed miRNAs in MB were up-regulated in comparison to normal CD133+ NSCs. This is in contrast to previous reports of general down-regulation of miRNA gene expression in primary human MB compared to normal adult and fetal cerebellum [33], [34], [36]. A possible explanation for this discrepancy lies with the varying differentiation status of each of the normal populations used for comparison. Accumulating evidence indicates that miRNA expression profiles reflect the state of cellular differentiation, due to the critical role of miRNAs in the regulation of the terminal differentiation of numerous cell types [24], [30], [55], [56]. Consistent with this, miRNA profiling studies performed in embryonic stem cells, embryoid bodies and day 11 mouse embryos indicated that both miRNA expression levels and the number of different miRNAs expressed increased as the maturation process progressed towards terminal differentiation [57]. Furthermore, recent studies have indicated that normal human adult cerebellum has higher expression levels of miRNAs when compared to pediatric cerebellum, consistent with developmentally regulated temporal expression of miRNAs in the brain [36]. Therefore, we suggest that the over-expression of miRNAs in MB observed in this study reflects our comparison of miRNA levels in MB to multipotent NSCs.

Clustering of human miRNA genes is common, with 38% of known miRNA genes residing in clusters of two or more separated by less than 5000 nucleotides [58], [59]. We found multiple miRNAs with significantly altered expression in MB mapped to identical chromosomal regions, including 14q32, which contains one of the largest known bipartite miRNA clusters in vertebrates, consisting of 47 miRNA genes in total [20], [60]. Previous studies reported that miRNAs mapping to this region are transcribed as a long polycistronic transcript, spanning approximately 210 kb of the mouse genome [60], [61]. Evolutionary conservation of clustered miRNA genes suggests an important common biological function, with clustered miRNAs shown to co-regulate identical targets or other components of the same pathway [62], [63]. Consistent with this, we identified several miRNAs mapping to 14q32 that were predicted to target the same down-regulated target genes. Thus, the identification of aberrantly expressed miRNA genes clustered in identical chromosomal locations suggests they may have a critical role in the coordinate regulation of target gene expression in MB.

Up-regulation of miRNAs mapping to 14q32 has been reported in other cancers including acute myeloid leukemia [64] and gastrointestinal stromal tumors [65]. Additionally, findings obtained in this study are consistent with those described by Northcott *et al* (2009) [33], where the up-regulation of several miRNAs mapping to 14q32 was observed in metastatic MB specimens with a neuronal differentiation signature. Given that a subset of primary MB specimens utilized in this study shared this signature, this suggests that the up-regulation of miRNAs mapping to 14q32 may be representative of metastatic disease. Despite this, the precise function of miRNAs mapping to 14q32 has not been elucidated. Expression of several miRNAs mapping to this region has been demonstrated throughout embryonic development in mice, progressively decreasing during postnatal development and ultimately becoming restricted to the adult brain [60], [61]. Gene ontology (GO) enrichment analysis performed on predicted targets of these miRNAs pointed to their roles in neurogenesis, cell motility and nervous system development [66]. Moreover, several genes of the MAPK signaling pathway predicted to be targeted by miRNAs mapping to 14q32 have been previously shown to be aberrantly expressed in Group C and D MB primary specimens [8]; for instance, the MAPK signaling genes, *FGF13*, *MAPK10* and *FGFR2*, were down-regulated in primary MB specimens. Thus, our findings suggest that the up-regulation of miRNAs mapping to 14q32 in MB may regulate signaling pathways that have previously implicated in MB pathogenesis.

Enrichment analysis of predicted mRNA targets of the miRNAs that were aberrantly expressed in MB identified several pathways of interest in MB pathogenesis including neuronal development pathways, axonal guidance and Reelin signaling in neurons. Axonal guidance signaling has been shown to regulate both neuronal migration and survival [67], with several studies identifying aberrant expression of this signaling pathway in a variety of different human tumor types (Reviewed in [68]). Similarly, Reelin signaling has been shown to play a critical role during embryonic brain development in the positioning and migration of Purkinje cells and subsequent expansion of granule cell precursors (GCPs) during early cerebellum development [69], [70]. Transgenic mouse studies have also indicated a role for Reelin in NSC proliferation, where an absence of *RELN* expression results in decreased NSC proliferation [71] and migration [71], [72]. IPA highlighted several putative miRNA-regulated signaling pathways containing the genes *PIK3CA* and *PIK3R1* as prominent in MB. Hyperactivation of Phosphoinositide 3-kinase (PI3K) signaling in several types of human cancers including MB is widely reported [73], [74], resulting in increased activity of the downstream effector Akt [75]. In turn, this promotes cancer cell growth [76], [77], invasion [78] and apoptosis resistance [79], [80] and thus PI3K and Akt have emerged as key targets for the development of novel anti-cancer therapeutics [81], [82]. *PIK3CA* expression is reportedly up-regulated in primary MB [83], and PI3K signaling is critical to normal GCP and MB cell proliferation [74], [83], [84]. Moreover, activation of PI3K signaling is essential for the proliferation of MB BTSCs residing in the perivascular niche following irradiation [85], suggesting that its targeted inhibition might impair the tumor resistance to radiation therapy. Thus, our findings suggest that the aberrant expression of miRNAs in MB may be involved in the regulation of critical pathways involved in embryonic and neuronal development, in addition to cell proliferation pathways previously linked to MB pathogenesis.

Integrative analysis of miRNA and mRNA gene expression is a robust and valuable approach for the identification of computationally predicted target genes in MB. Computational prediction of “true” miRNA targets is challenging, largely due to the context-dependent nature of post-transcriptional regulation resulting in a significant proportion of false-positive predicted miRNA/mRNA interactions [45], [86]. The approach taken in our study aimed to reduce the proportion of false-positive predictions, by integrating target gene predictions with gene expression profiles to select for inversely-correlated, functional miRNA/mRNA relationships. Whilst little is known about the role of miR-935-regulated target genes *KIAA0232* and *ZFAND6*, *SLC5A3* plays an important role in mammalian osmoregulation [87]. *SLC5A3* is a widely expressed sodium/myo-inositol (MI) co-transporter, functioning to maintain intracellular MI levels [88]. Alterations in MI levels have been suggested to affect phosphatidylinositol synthesis [89], with altered levels of Phosphatidylinositol 3-phosphate (*PI3P*) and Phosphatidylinositol-4,5-bisphosphate (*PIP2*) possibly linked to aberrant PI3K signaling [90]. Taken together, experimental validation of several miR-935 regulated target genes in MB cells confirmed the validity of this approach, suggesting that predicted miRNA/mRNA interactions obtained in our study may represent functional miRNA/mRNA networks in MB.

A comparison of previously published MB miRNA expression datasets reveals significant differences with respect to expression patterns of individual miRNAs. Previous studies compared miRNA expression in primary MB specimens to both adult and fetal normal human cerebellum, with discrepancies likely due to varied source and age of the cerebellum used for comparison as previously discussed [36]. Not surprisingly, deregulated miRNAs identified in our study differ from those previously identified as deregulated in MB, likely due to our comparison to normal control reference CD133+ NSCs. More specifically, comparison of the available miRNA data sets relative to adult and fetal human cerebellum with our data identified no significantly deregulated miRNAs as overlapping between the three studies (Figure S3). Additionally, miRNA expression levels in different studies were obtained using varied profiling platforms, previously identified as a source of miRNA expression variation in other reports [91]. Despite this, the identification of deregulated miRNAs specific to individual MB sub-types, as performed by Northcott *et al* (2009) [33], revealed comparable findings to those obtained in this study. In particular, several miRNAs mapping to 14q32, including miR-376c, miR-495 and miR-539, were identified as up-regulated in MB specimens defined by a neuronal differentiation signature (sub-type C as per [8]) [33]. This is consistent with our findings in which the majority of MB specimens were also characterized by either the neuronal differentiation (sub-type C) or mixed neuronal and photoreceptor gene signatures (sub-type DE). In addition, two miRNAs of the miR-106/-363 cluster mapping to chromosomal region Xq26.2 were specifically down-regulated in tumours characterized by a neuronal differentiation signature [33], consistent with the down-regulation of Xq26.2 miRNA, miR-106a* observed in this study. Therefore, future MB miRNA profiling studies should take into account potential inconsistencies between dataset comparisons due to both the platforms used and the source of normal reference RNA.

In summary, using an integrative approach we have identified aberrant miRNA-regulated networks that may be involved in the transformation of normal NSCs to BTSCs. These data provide a platform for future investigations aimed at characterizing the functional significance of these networks in MB pathogenesis.

## Supporting Information

Figure S1 **Range of Ct values of EC reference genes across all samples.** *MammU6* and *RNU48* are the most abundantly expressed EC reference genes. All samples were included in this analysis except for one primary MB sample, due to this sample failing with Pool B TLDA card.
(EPS)

Figure S2 **Overlap of deregulated miRNAs in primary MB specimens when compared to both CD133+ NSCs and CD133− NPCs.** miRNA expression in normal CD133+ NSCs/CD133− NPCs was determined from averaging log_2_(2^−ΔCt^) transformed miRNA values of CD133+ NSCs/CD133− NPCs from both hES3 and MEL1 ESC lines.
(EPS)

Figure S3 **Comparison analyses of deregulated miRNAs identified in primary MB specimens relative to previously published studies of Ferretti et al., (2009) and Northcott et al. (2009).** Venn diagrams were constructed containing significantly differentially expressed miRNAs identified in primary specimens relative to CD133+ NSCs from this study and all deregulated miRNAs (p<0.05) in primary MB specimens relative to (**A**) both adult and fetal human cerebellum and (**B**) human fetal cerebellum alone.
(EPS)

Table S1 **Repeated pair-wise correlation analysis between Endogenous control (EC) genes and Bestkeeper Index (BI).** Highest ranked correlations for *RNU6B* (r = 0.897), *MammU6* (r = 0.866), *RNU43* (r = 0.792) and *RNU48* (r = 0.723). Three candidate pairs of EC genes were identified with similar levels of correlation to each other and to BI, including *RNU6B/RNU43*, *MammU6/RNU48* and *RNU48/RNU24*.
(DOC)

Table S2 **Significantly up- and down-regulated miRNAs in primary MB specimens and/or MB cell lines relative to CD133+ NSCs.** miRNAs sorted according to their chromosomal location. miRNA expression in normal CD133+ NSCs was determined from averaging log_2_(2^−ΔCt^) transformed miRNA values of CD133+ NSCs from both hES3 and MEL1 ESC lines.
(DOC)

Table S3 **Significantly up- and down-regulated miRNAs in primary MB specimens relative to CD133− NPCs.** miRNAs are sorted according to their chromosomal location. miRNA expression in normal CD133+ NSCs was determined from averaging log_2_(2^−ΔCt^) transformed miRNA values of CD133+ NSCs from both hES3 and MEL1 ESC lines.
(DOC)

Table S4 **Down-regulated putative mRNA target genes of up-regulated miRNAs in MB.** All predicted miRNA target genes listed in the table were down-regulated in primary MB specimens, relative to CD133+ NSCs. All target genes listed were included in IPA pathway enrichment analysis.
(DOC)

Table S5 **Up-regulated putative mRNA target genes of down-regulated miRNAs in MB.** All predicted miRNA target genes listed in the table were up-regulated in primary MB specimens, relative to CD133+ NSCs. All target genes listed were included in IPA pathway enrichment analysis.
(DOC)

Table S6 **Enrichment analysis of the top 30 IPA pathway curated gene sets using putative mRNA target genes of up-regulated and down-regulated miRNAs in primary MB specimens relative to CD133+ NSCs.**
(DOC)

Table S7 **Enrichment analysis of the top 30 IPA pathway curated gene sets using putative mRNA target genes of up-regulated and down-regulated miRNAs in primary MB specimens relative to CD133− NPCs.**
(DOC)
