# Supplementary material for: Integrated Analysis of miRNA and mRNA Expression in Childhood Medulloblastoma Compared with Neural Stem Cells
Source: PLoS One. 2011 Sep 9;6(9):e23935. doi: 10.1371/journal.pone.0023935 (PMC3170291; doi:10.1371/journal.pone.0023935)
Supplement: Table S1 — Repeated pair-wise correlation analysis between Endogenous control (EC) genes and Bestkeeper Index (BI). Highest ranked correlations for RNU6B (r = 0.897), MammU6 (r = 0.866), RNU43 (r = 0.792) and RNU48 (r = 0.723). Three candidate pairs of EC genes were identified with similar levels of correlation to each other and to BI, including RNU6B/RNU43, MammU6/RNU48 and RNU48/RNU24. (DOC) [file pone.0023935.s004.doc]

| vs. | *MammU6* | *RNU43* | *RNU48* | *RNU24* | *RNU44* | *RNU6B* |
| --- | --- | --- | --- | --- | --- | --- |
| *RNU43* | 0.498 | - | - | - | - | - |
| p-value | 0.035 | - | - | - | - | - |
| *RNU48* | 0.765 | 0.23 | - | - | - | - |
| p-value | 0.001 | 0.356 | - | - | - | - |
| *RNU24* | 0.653 | 0.351 | 0.77 | - | - | - |
| p-value | 0.003 | 0.153 | 0.001 | - | - | - |
| *RNU44* | 0.268 | 0.035 | 0.389 | 0.348 | - | - |
| p-value | 0.283 | 0.890 | 0.110 | 0.158 | - | - |
| *RNU6B* | 0.677 | 0.862 | 0.45 | 0.446 | 0.295 | - |
| p-value | 0.002 | 0.001 | 0.060 | 0.064 | 0.236 | - |
|  |  |  |  |  |  |  |
| BestKeeper vs. | *MammU6* | *RNU43* | *RNU48* | *RNU24* | *RNU44* | *RNU6B* |
| coeff. of corr. [r] | 0.866 | 0.792 | 0.723 | 0.712 | 0.388 | 0.897 |
| p-value | 0.001 | 0.001 | 0.001 | 0.001 | 0.112374 | 0.001 |
