# Supplementary material for: Integrated Analysis of miRNA and mRNA Expression in Childhood Medulloblastoma Compared with Neural Stem Cells
Source: PLoS One. 2011 Sep 9;6(9):e23935. doi: 10.1371/journal.pone.0023935 (PMC3170291; doi:10.1371/journal.pone.0023935)
Supplement: Table S2 — Significantly up- and down-regulated miRNAs in primary MB specimens and/or MB cell lines relative to CD133+ NSCs. miRNAs sorted according to their chromosomal location. miRNA expression in normal CD133+ NSCs was determined from averaging log2(2−ΔCt) transformed miRNA values of CD133+ NSCs from both hES3 and MEL1 ESC lines. (DOC) [file pone.0023935.s005.doc]

| **miRNA** | **Chromosomal location** | **Expression in MB vs CD133+ NSCs** | **p value (MB primary specimens vs CD133pos)** | **p value (MB cell lines vs CD133pos)** |
| --- | --- | --- | --- | --- |
| hsa-miR-938 | 10p11.23 | Down | 0.0008 | 0.0002 |
| hsa-miR-483-5p | 11p15.5 | Down | 0.0091 | 0.0058 |
| hsa-miR-16 | 13q14.2/ 3q25.33 | Up | 0.0041 | 0.0875 |
| hsa-miR-17* | 13q31.3 | Up | 0.0180 | 0.0056 |
| hsa-miR-127-3p | 14q32.2 | Up | 0.0001 | 0.0596 |
| hsa-miR-539 | 14q32.31 | Up | <0.0001 | 0.0991 |
| hsa-miR-495 | 14q32.31 | Up | <0.0001 | 0.0168 |
| hsa-miR-409-3p | 14q32.31 | Up | 0.0001 | 0.0358 |
| hsa-miR-494 | 14q32.31 | Up | 0.0012 | 0.0198 |
| hsa-miR-376c | 14q32.31 | Up | 0.0014 | 0.0263 |
| hsa-miR-379 | 14q32.31 | Up | 0.0019 | 0.0858 |
| hsa-miR-376a | 14q32.31 | Up | 0.0073 | 0.0818 |
| hsa-miR-323-3p | 14q32.31 | Up | 0.0077 | 0.0480 |
| hsa-miR-203 | 14q32.33 | Up | 0.0004 | 0.0301 |
| hsa-miR-744* | 17p12 | Up | 0.0001 | 0.2466 |
| hsa-miR-193a-5p | 17q11.2 | Up | 0.0001 | 0.9831 |
| hsa-miR-144* | 17q11.2 | Up | 0.0069 | 0.7949 |
| hsa-miR-10a | 17q21.32 | Down | 0.0007 | 0.0179 |
| hsa-miR-21* | 17q23.1 | Up | 0.0025 | 0.1903 |
| hsa-miR-338-3p | 17q25.3 | Up | 0.0043 | 0.0608 |
| hsa-miR-133a | 18q11.2 / 20q13.33 | Down | 0.2922 | 0.0006 |
| hsa-miR-373 | 19q13.42 | Down | <0.0001 | 0.0081 |
| hsa-miR-935 | 19q13.42 | Down | 0.0002 | 0.0137 |
| hsa-miR-517a | 19q13.42 | Down | 0.0016 | 0.0135 |
| hsa-miR-92b* | 1q22 | Down | 0.0050 | 0.0140 |
| hsa-miR-185 | 22q11.21 | Up | 0.0002 | 0.0215 |
| hsa-miR-425* | 3p21.31 | Up | 0.0016 | 0.3689 |
| hsa-miR-566 | 3p21.31 | Down | 0.0233 | 0.0012 |
| hsa-miR-302b* | 4q25 | Down | 0.0005 | 0.0179 |
| hsa-miR-302d* | 4q25 | Down | 0.0049 | 0.0442 |
| hsa-miR-874 | 5q31.2 | Up | 0.0080 | 0.5027 |
| hsa-miR-143 | 5q32 | Up | <0.0001 | 0.5838 |
| hsa-miR-145 | 5q32 | Up | 0.0016 | 0.2848 |
| hsa-miR-146a | 5q34 | Up | <0.0001 | 0.0417 |
| hsa-miR-219-1-3p | 6p21.32 | Down | 0.0002 | 0.0010 |
| hsa-miR-219-5p | 6p21.32/ 9q34.11 | Down | 0.0008 | 0.0093 |
| hsa-miR-25 | 7q22.1 | Up | 0.0012 | 0.0389 |
| hsa-miR-182 | 7q32.2 | Up | 0.2111 | 0.0055 |
| hsa-miR-183 | 7q32.2 | Up | 0.2244 | 0.0083 |
| hsa-miR-183* | 7q32.2 | Up | 0.5315 | 0.0061 |
| hsa-miR-875-5p | 8q22.2 | Down | 0.0026 | 0.0277 |
| hsa-miR-219-2-3p | 9q34.11 | Down | 0.0003 | 0.0154 |
| hsa-miR-126 | 9q34.3 | Up | <0.0001 | 0.7110 |
| hsa-miR-126* | 9q34.3 | Up | <0.0001 | 0.7514 |
| hsa-miR-223 | Xq12 | Up | 0.0052 | 0.2494 |
| hsa-miR-361-3p | Xq21.2 | Up | 0.0009 | 0.8435 |
| hsa-miR-652 | Xq23 | Up | 0.0001 | 0.0039 |
| hsa-miR-106a* | Xq26.2 | Down | 0.0100 | 0.0231 |
| hsa-miR-363 | Xq26.2 | Down | 0.0290 | 0.0009 |
| hsa-miR-504 | Xq26.3 | Down | 0.0003 | <0.0001 |
