# Supplementary material for: Integrated Analysis of miRNA and mRNA Expression in Childhood Medulloblastoma Compared with Neural Stem Cells
Source: PLoS One. 2011 Sep 9;6(9):e23935. doi: 10.1371/journal.pone.0023935 (PMC3170291; doi:10.1371/journal.pone.0023935)
Supplement: Table S3 — Significantly up- and down-regulated miRNAs in primary MB specimens relative to CD133− NPCs. miRNAs are sorted according to their chromosomal location. miRNA expression in normal CD133+ NSCs was determined from averaging log2(2−ΔCt) transformed miRNA values of CD133+ NSCs from both hES3 and MEL1 ESC lines. (DOC) [file pone.0023935.s006.doc]

| **miRNA** | **Chromosomal location** | **p value** | **FDR adjusted p value** | **Fold Change (log2)** |
| --- | --- | --- | --- | --- |
| **UP-REGULATED** |  |  |  |  |
| hsa-miR-605 | 10q21.1 | 0.0086 | 0.0779 | 3.12 |
| hsa-miR-15a | 13q14.2 | <0.0001 | 0.0002 | 4.40 |
| hsa-miR-127-3p | 14q32.2 | <0.0001 | 0.0009 | 6.53 |
| hsa-miR-370 | 14q32.2 | <0.0001 | 0.0014 | 7.55 |
| hsa-miR-136* | 14q32.2 | 0.0001 | 0.0039 | 6.54 |
| hsa-miR-493 | 14q32.2 | 0.0004 | 0.0100 | 3.51 |
| hsa-miR-433 | 14q32.2 | 0.0037 | 0.0451 | 3.24 |
| hsa-miR-337-5p | 14q32.2 | 0.0047 | 0.0534 | 3.26 |
| hsa-miR-432 | 14q32.2 | 0.0057 | 0.0617 | 3.75 |
| hsa-miR-382 | 14q32.31 | <0.0001 | 0.0006 | 8.16 |
| hsa-miR-379 | 14q32.31 | 0.0001 | 0.0037 | 5.04 |
| hsa-miR-134 | 14q32.31 | 0.0002 | 0.0064 | 4.77 |
| hsa-miR-539 | 14q32.31 | 0.0004 | 0.0093 | 4.42 |
| hsa-miR-494 | 14q32.31 | 0.0005 | 0.0110 | 4.22 |
| hsa-miR-656 | 14q32.31 | 0.0007 | 0.0121 | 4.63 |
| hsa-miR-410 | 14q32.31 | 0.0016 | 0.0264 | 3.66 |
| hsa-miR-889 | 14q32.31 | 0.0017 | 0.0269 | 4.02 |
| hsa-miR-409-3p | 14q32.31 | 0.0020 | 0.0298 | 5.50 |
| hsa-miR-655 | 14q32.31 | 0.0058 | 0.0621 | 4.08 |
| hsa-miR-758 | 14q32.31 | 0.0064 | 0.0649 | 3.57 |
| hsa-miR-376a | 14q32.31 | 0.0080 | 0.0748 | 4.48 |
| hsa-miR-543 | 14q32.31 | 0.0083 | 0.0763 | 5.48 |
| hsa-miR-411 | 14q32.31 | 0.0088 | 0.0779 | 4.77 |
| hsa-miR-203 | 14q32.33 | 0.0006 | 0.0121 | 5.87 |
| hsa-miR-195 | 17p13.1 | 0.0001 | 0.0035 | 3.48 |
| hsa-miR-22 | 17p13.3 | 0.0003 | 0.0093 | 6.65 |
| hsa-miR-451 | 17q11.2 | <0.0001 | 0.0000 | 10.60 |
| hsa-miR-193a-3p | 17q11.2 | 0.0016 | 0.0265 | 4.73 |
| hsa-miR-142-3p | 17q22 | <0.0001 | 0.0004 | 9.44 |
| hsa-miR-142-5p | 17q22 | 0.0023 | 0.0326 | 3.25 |
| hsa-miR-21 | 17q23.1 | 0.0029 | 0.0392 | 3.57 |
| hsa-miR-639 | 19p13.12 | 0.0003 | 0.0091 | 3.85 |
| hsa-miR-27a | 19p13.13 | 0.0007 | 0.0121 | 3.86 |
| hsa-miR-150 | 19q13.33 | 0.0004 | 0.0095 | 8.85 |
| hsa-miR-296-3p | 20q13.32 | <0.0001 | 0.0014 | 3.12 |
| hsa-miR-886-3p | 5q31.1 | <0.0001 | 0.0002 | 9.32 |
| hsa-miR-886-5p | 5q31.1 | <0.0001 | 0.0009 | 7.64 |
| hsa-miR-145* | 5q32 | 0.0001 | 0.0046 | 5.17 |
| hsa-miR-146a | 5q34 | <0.0001 | 0.0002 | 7.79 |
| hsa-miR-29b | 7q32.3 | 0.0003 | 0.0093 | 4.41 |
| hsa-miR-29a* | 7q32.3 | 0.0061 | 0.0646 | 3.72 |
| hsa-miR-455-5p | 9q32 | 0.0005 | 0.0110 | 3.92 |
| hsa-miR-126 | 9q34.3 | 0.0072 | 0.0700 | 5.96 |
| hsa-miR-126* | 9q34.3 | 0.0073 | 0.0705 | 6.84 |
| **DOWN-REGULATED** |  |  |  |  |
| hsa-miR-615-3p | 12q13.13 | 0.0002 | 0.0059 | -5.08 |
| hsa-miR-10a | 17q21.32 | 0.0005 | 0.0110 | -5.44 |
| hsa-miR-935 | 19q13.42 | 0.0003 | 0.0086 | -8.76 |
| hsa-miR-10b | 2q31.1 | 0.0093 | 0.0808 | -5.76 |
| hsa-miR-219-1-3p | 6p21.32 | 0.0006 | 0.0112 | -4.18 |
| hsa-miR-219-5p | 6p21.32/ 9q34.11 | 0.0002 | 0.0078 | -5.91 |
| hsa-miR-219-2-3p | 9q34.11 | 0.0002 | 0.0078 | -7.83 |
| hsa-miR-18b* | Xq26.2 | 0.0042 | 0.0495 | -3.75 |
| hsa-miR-504 | Xq26.3 | 0.0001 | 0.0035 | -6.01 |
