# Supplementary material for: Integrated Analysis of miRNA and mRNA Expression in Childhood Medulloblastoma Compared with Neural Stem Cells
Source: PLoS One. 2011 Sep 9;6(9):e23935. doi: 10.1371/journal.pone.0023935 (PMC3170291; doi:10.1371/journal.pone.0023935)
Supplement: Table S4 — Down-regulated putative mRNA target genes of up-regulated miRNAs in MB. All predicted miRNA target genes listed in the table were down-regulated in primary MB specimens, relative to CD133+ NSCs. All target genes listed were included in IPA pathway enrichment analysis. (DOC) [file pone.0023935.s007.doc]

**Table S4:** **Down-regulated** **putative mRNA target genes of up-regulated miRNAs** **in MB.**

| **miRNA** | **Direct target genes** |
| --- | --- |
| hsa-miR-145 | ADD3, SOX9, QKI, MEST, SMAD3, NEDD9, DUSP6, AKAP12, GRB10, SNX24, GINS3, KLF3, HIC2, ELMO1, PSAT1, SLC7A8, NR4A2, PGD, H2AFX, FUT9, CDH2, PSD3, SNX15, MDFIC, CYR61, LMNB2, RBM47, CELSR1, MEIS1, P4HA1, ZNF516, TSPAN6, FZD7, SULF1, SLC16A2, MAF, NCALD, EFNB3, KLF4, PTPN3 |
| hsa-miR-495 | CTNND2, DDIT4, EVI1, GAS1, MAPK10, ADAMTS3, PLCH1, SOX13, CYB5B, COL21A1, RAN, TMEM97, EPB41L4B, ZFHX3, PDLIM5, NMT2, MMRN1, SCD, HNRNPUL2, HSDL2, CYP26B1, MSX1, EPHB2, LPHN2, NOTCH1, SNRPB, ID1, EMX2, RPGRIP1L, SHMT2, ELAVL1, TOX, NEBL, SHANK2 |
| hsa-miR-203 | NUDT21, ADK, HNRNPUL2, CCNG1, D4S234E, SEMA5A, GALNT10, GPC4, PPAP2B, RPGRIP1L, RBM47, PSD3, ADAMTS8, PTPN3, CDH10, TOX3, SFRP1, BIRC5, GLI3, NMNAT2, ID4, LMNB2, ANP32E, PXDN, GALNT7, STON1, SNX24, CTSC, RAN, EN2, HTR2C, SMAD3 |
| hsa-miR-323-3p | PCDH8, LMO3, CDH6, GAD1, ZFHX3, FUT9, SMAD3, NR4A2, ARL4C, HSPA4, ZNF516, LIPG, HMGCS1, WNT5A, MAPK1IP1L, NLGN4X, PPAP2B, TOX3, EPHA3, ASCL1, GHR, MYH10, PRDM4, KLF3 |
| hsa-miR-539 | GALNT7, FGF13, RRM2, ELAVL1, RFC3, ABCC4, SOX13, LIPG, DSCC1, HS2ST1, MYCL1, FBXL7, PHB2, HIC2, KIF11, DEPDC1, ADM, NR4A2, PDLIM5, ZIC3, CDC25A |
| hsa-miR-494 | HMGCS1, NMNAT2, ZFHX3, GALNT7, GLI3, GULP1, TACC2, CKS1B, FGFR2, SFRS7, EN2, P4HA1, SHANK2, CHN1, ATF3, ERLIN2, MAF, CTNND2, HS2ST1, LMNB2 |
| hsa-miR-376c | SASH1, BAZ1A, EN2, GULP1, OPCML, GPM6B, OPN3, CALB1, NR2F2, KPNA2, RIMS3, TOX, PPAP2B, MSX1, TIAM1, SHANK2, NR4A2 |
| hsa-miR-143 | SLC7A11, GLI3, ITGA6, HTR2C, ADD3, MAF, FAM60A, ABCC4, PSD3, SLC16A2, NUAK2, NFATC1, PDIA6, FLJ10357, SMAD3 |
| hsa-miR-223 | ECT2, SEPT10, FAM46A, CENPN, RRAS2, NUP210, SACS, MYH10, ZFHX3, FGFR2, AK2, SC4MOL, FAT, RAB8B, TMEM47 |
| hsa-miR-409-3p | NUDT21, MSH6, SALL1, PPAP2B, STON1, SASH1, NAV2, NR2F2, NR4A2, SRI, TCF3, HN1L, SEPHS1 |
| hsa-miR-338-3p | LMO3, PPP1R1A, AKAP12, SALL1, EVI1, MAF, CSDA, FGFR2, MSN, BAMBI, RRM1, GRIN1 |
| hsa-miR-376a | SLC7A11, TIAM1, PDIA6, RBMS1, EML1, POLQ, PRKD1, SALL1, PLAG1, NLGN4X, ZFHX3, EN2 |
| hsa-miR-146a | NRAS, SLC2A3, GALNT10, EDNRB, LIN28 |
| hsa-miR-361-3p | TMEM2, SOX13, STC2 |
| hsa-miR-193a-5p | FADS1, NTRK2, HIC2 |
| hsa-miR-379 | CCNB1, YARS |
