# Supplementary material for: Integrated Analysis of miRNA and mRNA Expression in Childhood Medulloblastoma Compared with Neural Stem Cells
Source: PLoS One. 2011 Sep 9;6(9):e23935. doi: 10.1371/journal.pone.0023935 (PMC3170291; doi:10.1371/journal.pone.0023935)
Supplement: Table S5 — Up-regulated putative mRNA target genes of down-regulated miRNAs in MB. All predicted miRNA target genes listed in the table were up-regulated in primary MB specimens, relative to CD133+ NSCs. All target genes listed were included in IPA pathway enrichment analysis. (DOC) [file pone.0023935.s008.doc]

| **miRNA** | **Direct target genes** |
| --- | --- |
| hsa-miR-373 | TGFBR2, OXR1, MSL-1, RSBN1, UBE2B, GPM6A, CD44, SNRK, MYT1L, GLCE, ARHGEF3, DCX, SPOP, ARID4A, RTN1, NECAP1, FOXO3, TIMP3, CMPK1, INTS6, RALGDS, MLL, ZNF148, RAD23B, SS18L1, ZFPM2, RNF6, KIAA0240, MTMR3, OSTM1, NHLH2, SMAD2, FBXO11, GNB5, CCND2, NFIB, |
| hsa-miR-219-5p | ZNF238, TSC22D2, KIAA0240, CCDC28A, CC2D1A, TXNDC13, TGFBR2, ACOX1, DNAJC6, EYA2, PKNOX1, RNF6, LPGAT1, LAPTM4A, GLCE, SNRK, AKAP13, KCNJ2, SCARB2, ZNF148, UBE3A, NECAP1, SATB1, KIAA0182, RYBP, PIP5K1C, DMXL1, CBFA2T3, FBXO41, LIN7C, TOB2, RAD23B, ARHGEF9 |
| hsa-miR-935 | SLC5A3, RELN, KIAA0232, TBC1D9, MYT1, ZFAND6, LMO2, TMEM1, SCARB2, LETMD1, MBNL2, NMT1, ARHGEF3, APC, OSTM1, LBH, FBXO28, ZFAND5, FBXO11, NCAM1, CACNA2D2, KHDRBS2, PCTK2, SPOCK2, STX6, KIAA0247, CREBZF |
| hsa-miR-10a | ELOVL2, NCOR2, MAP4K4, FNBP1L, RAP2A, PIK3CA, PSCD1, MTMR3, MED1, SMAD2, KIAA1462, LANCL1, BAZ2B, H3F3B, STARD13, ZFAND5, SCARB2, MYT1L, NFAT5, MTF2, ESRRG, INHBB, UBXD7, BCL2L2, ANKRD12, KIAA0247 |
| hsa-miR-219-2-3p | RSBN1, SERINC1, TSNAX, SSTR2, GOLGA8A, C7, ENC1, PMEPA1, SOX4, ARHGEF9, ATP8A1, FRMD4B, SATB1, BMI1, SCN3B, WASL, NFIB, DCX, C14orf101 |
| hsa-miR-504 | CEP170, PRKAR2A, MYT1L, H3F3B, ZFAND5, DCX, PIK3R1, MAPRE2, RBM9, TNRC4, PSMD3 |
| hsa-miR-219-1-3p | PDZD2, GNB5, STAG2, NF1, FN1, ARHGEF9, DCLK1, SOX4 |
