# Supplementary material for: Integrated Analysis of miRNA and mRNA Expression in Childhood Medulloblastoma Compared with Neural Stem Cells
Source: PLoS One. 2011 Sep 9;6(9):e23935. doi: 10.1371/journal.pone.0023935 (PMC3170291; doi:10.1371/journal.pone.0023935)
Supplement: Table S6 — Enrichment analysis of the top 30 IPA pathway curated gene sets using putative mRNA target genes of up-regulated and down-regulated miRNAs in primary MB specimens relative to CD133+ NSCs. (DOC) [file pone.0023935.s009.doc]

| **Ingenuity Canonical Pathways** | **p value** | **Genes** |
| --- | --- | --- |
| Molecular Mechanisms of Cancer | <0.0001 | NF1, PIK3R1, APC, ARHGEF3, WNT5A, PRKAR2A, PRKD1, NOTCH1, SMAD3, PIK3CA, TCF3, CCND2, SMAD2, RALGDS, FZD7, ARHGEF12, TGFBR2, RRAS2, RAP2A, PRKACB, NRAS, CDC25A, MAPK10 |
| Colorectal Cancer Metastasis Signaling | <0.0001 | PIK3R1, APC, WNT5A, PRKAR2A, SMAD3, PIK3CA, TCF3, SMAD2, RALGDS, MSH6, FZD7, TGFBR2, RRAS2, PRKACB, NRAS, GNB5, BIRC5, MAPK10 |
| Ovarian Cancer Signaling | <0.0001 | PIK3R1, APC, FZD7, RRAS2, WNT5A, PRKAR2A, TCF3, PIK3CA, NRAS, PRKACB, CD44, MSH6 |
| Reelin Signaling in Neurons | <0.0001 | PIK3R1, ARHGEF12, ARHGEF3, ITGA6, PIK3CA, RELN, DCX, ARHGEF9, MAPK10 |
| Axonal Guidance Signaling | 0.0001 | PIK3R1, GLI3, WNT5A, PRKAR2A, PRKD1, PIK3CA, WASL (includes EG:8976), EPHA3, SEMA5A, NFATC1, FZD7, EFNB3, ARHGEF12, RRAS2, NFAT5, EPHB2, PRKACB, NRAS, GNB5, NTRK2, SHANK2 |
| IGF-1 Signaling | 0.0002 | PIK3R1, RRAS2, PRKAR2A, PIK3CA, GRB10, NRAS, PRKACB, FOXO3, CYR61 |
| Human Embryonic Stem Cell Pluripotency | 0.0002 | PIK3R1, APC, FZD7, FGFR2, TGFBR2, WNT5A, SMAD3, TCF3, PIK3CA, SMAD2, NTRK2 |
| PPARα/RXRα Activation | 0.0003 | NCOR2, RRAS2, TGFBR2, PRKAR2A, MED1, SMAD3, NRAS, PRKACB, MAP4K4, SMAD2, ACOX1, GHR |
| Regulation of IL-2 Expression in Activated and Anergic T Lymphocytes | 0.0003 | NFATC1, RRAS2, TGFBR2, NFAT5, SMAD3, NRAS, SMAD2, MAPK10 |
| PTEN Signaling | 0.0005 | PIK3R1, FGFR2, RRAS2, TGFBR2, PIK3CA, NRAS, FOXO3, NTRK2, GHR |
| Pancreatic Adenocarcinoma Signaling | 0.0005 | PIK3R1, TGFBR2, NOTCH1, SMAD3, PIK3CA, SMAD2, BIRC5, RALGDS, MAPK10 |
| Germ Cell-Sertoli Cell Junction Signaling | 0.0007 | PIK3R1, RRAS2, TGFBR2, ITGA6, PIK3CA, RAB8B, NRAS, WASL (includes EG:8976), PPAP2B, MAPK10, CDH2 |
| Actin Cytoskeleton Signaling | 0.0007 | MSN, MYH10, PIK3R1, APC, PIK3CA, FGF13, WASL (includes EG:8976), ARHGEF12, RRAS2, FN1, TIAM1, NRAS, PIP5K1C |
| Protein Kinase A Signaling | 0.0010 | PPP1R10, MYH10, GLI3, PRKAR2A, PRKD1, SMAD3, ADD3, AKAP12, TCF3, H3F3B, NFATC1, AKAP13, TGFBR2, NFAT5, PRKACB, GNB5 |
| RAR Activation | 0.0010 | NCOR2, PIK3R1, PRKAR2A, MED1, PRKD1, SMAD3, PIK3CA, PRKACB, SMAD2, NR2F2, MAPK10 |
| Breast Cancer Regulation by Stathmin1 | 0.0010 | PPP1R10, PIK3R1, ARHGEF12, RRAS2, ARHGEF3, PRKAR2A, PRKD1, PIK3CA, NRAS, PRKACB, GNB5, RB1CC1 |
| Wnt/β-catenin Signaling | 0.0012 | APC, FZD7, TGFBR2, SOX9, WNT5A, SOX4, TCF3, SFRP1, SOX13, CD44, CDH2 |
| Glucocorticoid Receptor Signaling | 0.0013 | PIK3R1, MED1, SMAD3, PIK3CA, SMAD2, NFATC1, NCOR2, HSPA4, TGFBR2, RRAS2, NFAT5, PRKACB, NRAS, MAPK10 |
| Glioma Invasiveness Signaling | 0.0014 | PIK3R1, TIMP3, RRAS2, PIK3CA, NRAS, CD44 |
| FcγRIIB Signaling in B Lymphocytes | 0.0018 | PIK3R1, RRAS2, PIK3CA, NRAS, MAPK10 |
| Synaptic Long Term Potentiation | 0.0019 | PPP1R10, PPP1R1A, RRAS2, GRIN1, PRKAR2A, PRKD1, NRAS, PRKACB |
| Insulin Receptor Signaling | 0.0021 | PPP1R10, PIK3R1, RRAS2, PRKAR2A, PIK3CA, GRB10, NRAS, PRKACB, FOXO3 |
| Renin-Angiotensin Signaling | 0.0022 | PIK3R1, RRAS2, PRKAR2A, PRKD1, PIK3CA, NRAS, PRKACB, MAPK10 |
| fMLP Signaling in Neutrophils | 0.0025 | NFATC1, PIK3R1, RRAS2, NFAT5, PRKD1, PIK3CA, NRAS, GNB5 |
| CDK5 Signaling | 0.0027 | PPP1R10, RRAS2, ITGA6, PRKAR2A, NRAS, PRKACB, NTRK2 |
| PAK Signaling | 0.0031 | PIK3R1, RRAS2, PIK3CA, NRAS, EPHA3, WASL (includes EG:8976), MAPK10 |
| Sonic Hedgehog Signaling | 0.0032 | GLI3, PRKAR2A, PRKACB, CCNB1 |
| NF-κB Signaling | 0.0035 | PIK3R1, FGFR2, RRAS2, TGFBR2, PIK3CA, NRAS, PRKACB, MAP4K4, NTRK2, GHR |
| SAPK/JNK Signaling | 0.0042 | NFATC1, PIK3R1, RRAS2, PIK3CA, NRAS, MAP4K4, MAPK10 |
| Gα12/13 Signaling | 0.0042 | PIK3R1, CDH10, RRAS2, PIK3CA, NRAS, CDH6, MAPK10, CDH2 |
