# Supplementary material for: Integrated Analysis of miRNA and mRNA Expression in Childhood Medulloblastoma Compared with Neural Stem Cells
Source: PLoS One. 2011 Sep 9;6(9):e23935. doi: 10.1371/journal.pone.0023935 (PMC3170291; doi:10.1371/journal.pone.0023935)
Supplement: Table S7 — Enrichment analysis of the top 30 IPA pathway curated gene sets using putative mRNA target genes of up-regulated and down-regulated miRNAs in primary MB specimens relative to CD133− NPCs. (DOC) [file pone.0023935.s010.doc]

| **Ingenuity Canonical Pathways** | **p value** | **Molecules** |
| --- | --- | --- |
| Wnt/β-catenin Signaling | <0.0001 | PPP2R1B, WIF1, WNT5A, ACVR2A, WNT4, TCF3, CCND1, SOX5 (includes EG:6660), SFRP1, ACVR2B, CDH2, TCF4, TCF7L1, FZD7, FZD10, SOX9, JUN, TLE4, SOX2, ACVR1B |
| Axonal Guidance Signaling | <0.0001 | PIK3R1, WNT5A, WNT4, PRKD1, PAK7, ROBO1, FZD7, CDC42, FZD10, EFNB3, ARHGEF12, EFNB2, BAIAP2, NGFR, SRGAP3, PRKCB, VEGFA, NRP1, GLI3, PPP3CB, SEMA5A, PLXNA2, PDGFC, EPHA4, EPHB2, SEMA6A, PLXNA1, SHANK2, EPHB3 |
| Role of Macrophages, Fibroblasts and Endothelial Cells in Rheumatoid Arthritis | <0.0001 | PIK3R1, WIF1, WNT5A, WNT4, PRKD1, FZD10, FZD7, FOS, CREB5, NGFR, PRKCB, VEGFA, PPP3CB, TCF3, MAP2K6, CCND1, CEBPB, SFRP1, ROR2, TCF4, TCF7L1, FN1, JUN, PDGFC, PLCD1 |
| Molecular Mechanisms of Cancer | <0.0001 | PRKCB, PMAIP1, PIK3R1, ARHGEF3, WNT5A, PRKD1, NOTCH1, SMAD4, TCF3, MAP2K6, RASGRP1, CCND1, ARHGEF10, CDKN1A, PAK7, TCF4, CTNNA1, FZD7, FZD10, ARHGEF12, CDC42, FOS, JUN, CHEK1 |
| Factors Promoting Cardiogenesis in Vertebrates | <0.0001 | PRKCB, TCF4, TCF7L1, FZD7, FZD10, ACVR2A, PRKD1, SMAD4, TCF3, ACVR1B, ACVR2B |
| VDR/RXR Activation | 0.0002 | PRKCB, NCOR2, PRKD1, HOXA10, KLF4, CEBPB, CDKN1A, CALB1, HES1 |
| p53 Signaling | 0.0002 | TNFRSF10B, PMAIP1, PIK3R1, CCNG1, SNAI2, JUN, CCND1, CHEK1, CDKN1A, PLAGL1 |
| Human Embryonic Stem Cell Pluripotency | 0.0002 | PIK3R1, TCF4, TCF7L1, FZD7, FGFR2, FZD10, WNT5A, WNT4, SMAD4, PDGFC, TCF3, SOX2 |
| Ovarian Cancer Signaling | 0.0002 | PIK3R1, TCF4, TCF7L1, FZD7, FZD10, VEGFA, WNT5A, WNT4, PDGFC, TCF3, CCND1, MMP2 |
| Role of Osteoblasts, Osteoclasts and Chondrocytes in Rheumatoid Arthritis | 0.0002 | PIK3R1, WIF1, PPP3CB, WNT5A, WNT4, SMAD4, TCF3, MAP2K6, SFRP1, TCF4, TCF7L1, FZD7, FZD10, FOS, JUN, NGFR |
| ILK Signaling | 0.0002 | MYH10, PIK3R1, VEGFA, SNAI2, PPP2R1B, MAP2K6, CCND1, CDC42, FN1, FOS, JUN, PDGFC, CREB5, PPAP2B |
| Basal Cell Carcinoma Signaling | 0.0004 | TCF4, TCF7L1, FZD7, FZD10, GLI3, WNT5A, WNT4, TCF3 |
| Neurotrophin/TRK Signaling | 0.0004 | PIK3R1, CDC42, FOS, SPRY2, JUN, MAP2K6, CREB5, NGFR |
| Colorectal Cancer Metastasis Signaling | 0.0004 | PIK3R1, VEGFA, WNT5A, SMAD4, WNT4, TCF3, MMP2, CCND1, MMP16, TCF4, TCF7L1, FZD7, FZD10, FOS, JUN, PDGFC |
| Clathrin-mediated Endocytosis Signaling | 0.0007 | PIK3R1, CDC42, VEGFA, PPP3CB, HIP1, PDGFC, DNM3, EPHB2, MYO6, FGF13, DAB2, LDLR |
| HER-2 Signaling in Breast Cancer | 0.0009 | PRKCB, PIK3R1, CDC42, PRKD1, ERBB3, CCND1, MMP2, CDKN1A |
| GNRH Signaling | 0.0011 | PAK7, PRKCB, CDC42, FOS, JUN, PRKD1, DNM3, MAP2K6, CREB5, EGR1 |
| Glioblastoma Multiforme Signaling | 0.0014 | PIK3R1, FZD7, CDC42, FZD10, WNT5A, WNT4, PDGFC, TCF3, CCND1, PLCD1, CDKN1A |
| Renal Cell Carcinoma Signaling | 0.0021 | PAK7, PIK3R1, CDC42, SLC2A1, VEGFA, FOS, JUN |
| LPS-stimulated MAPK Signaling | 0.0025 | PRKCB, PIK3R1, CDC42, FOS, JUN, PRKD1, MAP2K6 |
| Notch Signaling | 0.0026 | NOTCH1, JAG1, NOTCH2, DTX4, HES1 |
| TGF-β Signaling | 0.0033 | FOS, JUN, ACVR2A, SMAD4, ACVR1B, INHBB, ACVR2B |
| Reelin Signaling in Neurons | 0.0038 | PIK3R1, ARHGEF12, ARHGEF3, ARHGEF10, RELN, DCX, CNR1 |
| mTOR Signaling | 0.0038 | PRKCB, PIK3R1, VEGFA, PPP2R1B, PRKD1, PDGFC, EIF4G3, RPS6KA2, PRKAB2, DDIT4 |
| HGF Signaling | 0.0041 | PRKCB, PIK3R1, CDC42, FOS, JUN, PRKD1, CCND1, CDKN1A |
| Acute Myeloid Leukemia Signaling | 0.0041 | PIK3R1, TCF4, TCF7L1, CCNA1, TCF3, MAP2K6, CCND1 |
| Role of Wnt/GSK-3β Signaling in the Pathogenesis of Influenza | 0.0041 | TCF4, TCF7L1, FZD7, FZD10, WNT5A, WNT4, TCF3 |
| Ephrin Receptor Signaling | 0.0045 | PAK7, EFNB3, CDC42, GRIN1, VEGFA, EFNB2, PDGFC, EPHA4, EPHB2, CREB5, EPHB3 |
| Role of Oct4 in Mammalian Embryonic Stem Cell Pluripotency | 0.0054 | KDM5B, SOX2, NR2F6, BMI1, NR2F2 |
| HIF1α Signaling | 0.0058 | PIK3R1, SLC2A1, VEGFA, JUN, PDGFC, SLC2A3, MMP2, MMP16 |
